# Deep early tumour shrinkage in metastatic upper tract urothelial carcinoma treated with enfortumab vedotin plus pembrolizumab

**DOI:** 10.1111/bju.70174

**Published:** 2026-02-12

**Authors:** Marie Semmler, Maurice Heimer, Can Aydogdu, Benazir Enzinger, Isabel Brinkmann, Frederik Kolligs, Gerald B. Schulz, Christian G. Stief, Philipp M. Kazmierczak, Jozefina Casuscelli, Lennert Eismann

**Affiliations:** ^1^ Department of Urology LMU University Hospital, LMU Munich Munich Germany; ^2^ Department of Radiology LMU University Hospital, LMU Munich Munich Germany

**Abbreviations**
CT: computed tomography
dETS: deep Early Tumour Shrinkage
EV + P: enfortumab vedotin plus pembrolizumab
FU: follow‐up
IQR: interquartile range
NR: not reached
OS: overall survival
PFS: progression‐free survival
(i)RECIST (1.1): (immunotherapy) Response Evaluation Criteria in Solid Tumours (version 1.1)
TB: tumour burden
(m)(UT)UC: (metastatic) (upper tract) urothelial carcinoma

For decades, tumour response to systemic therapy has been evaluated using the Response Evaluation Criteria in Solid Tumours (RECIST), later adapted to RECIST version 1.1 (RECIST 1.1), which were originally developed and validated in cohorts primarily treated with conventional chemotherapy [1, 2]. With the advent of immunotherapy, these criteria were adapted to iRECIST to account for phenomena such as pseudo‐progression [3].

However, in metastatic urothelial carcinoma (UC) systemic therapy has evolved beyond chemotherapy and immune checkpoint inhibition in recent years. The current standard of care combines the antibody–drug conjugate enfortumab vedotin with the immune checkpoint inhibitor pembrolizumab (EV + P) [4], a regimen that profoundly alters treatment dynamics.

Upper tract UC (UTUC), although included in pivotal trials such as EV‐302 (ClinicalTrials.gov identifier: NCT04223856), is increasingly recognised as a distinct tumour entity with relevant molecular, genetic and clinical differences from bladder cancer [5]. These features may influence both treatment response and radiological appearance.

Despite therapeutic advances, response assessment in both clinical trials and routine practice continues to rely predominantly on RECIST‐based imaging criteria. However, these size‐based metrics were not designed to capture the complex and potentially heterogeneous tumour responses induced by combined regimens such as EV + P, particularly in distinct entities such as metastatic UTUC (mUTUC). This raises uncertainty about whether RECIST 1.1 adequately reflects true biological treatment effects in this context and predicts clinical outcomes.

To address this gap, we investigated a case series of mUTUC, focusing on radiological response on CT and the corresponding oncological outcomes. Our aim was to evaluate the response in imaging of mUTUC to the introduced first‐line treatment EV + P in a real‐world cohort, not only applying RECIST 1.1 response criteria, but also in‐depth analysis using tumour burden (TB) evolution and organ‐specific dynamics.

In this retrospective study, patients with synchronous and metachronous mUTUC were included. Patient characteristics at baseline and follow‐up (FU) data were collected. Baseline‐staging using CT was performed prior to initiation of EV + P. FU imaging was performed every 3 months or at clinical discretion.

Imaging data including overall TB and dynamics of organ‐specific metastasis were evaluated by a board‐certified radiologist according to RECIST 1.1. Largest diameter of primary tumour, metastasis in lymph nodes, bones, liver and in other organ systems were assessed. Therefore, a representative lesion per organ system was chosen and changes in size were captured. Reduction of TB >50% on first FU imaging was defined as deep Early Tumour Shrinkage (dETS).

Overall survival (OS) and progression‐free survival (PFS) were estimated using the Kaplan–Meier method. A univariate analysis of prognostic value was performed using the log‐rank test. A *P* < 0.05 was considered statistically significant.

Imaging data of 16 patients who initiated treatment with EV + P before November 2024 were analysed. The patients’ median (interquartile range [IQR]) age was 73 (57–80) years, and 44% (7/16) of patients were female. The primary tumour was located in the renal pelvis in 69% (11/16) of cases and in the ureter in 31% (5/16) of cases. At treatment initiation, 69% (11/16) of patients had *de novo* metastatic disease, while 31% (5/16) of patients developed metastases secondarily. Multiple metastatic sites were present in 75% of cases. Metastases involved lymph nodes in 69%, liver in 19%, bone in 38%, and other organs in 38%. The median (IQR) time on EV + P was 8 (3–11) months. Overall, disease progression was observed in 56% (9/16) of patients based on RECIST 1.1, median (IQR) PFS was 11 (8–not reached [NR]) months; 44% of patients deceased during follow up. The median (IQR) OS was 13 (11–NR)months.

According to RECIST 1.1, objective response at the first FU was 69% (11/16); however, no statistically significant effect of early radiological response regarding PFS (*P* = 0.7) and OS (*P* = 0.6) could be determined (Fig. 1A,C). Median (IQR) dynamics at first FU imaging revealed a 39% (25–55%) TB reduction and a median (IQR) reduction in the primary lesion of 16% (11–30%). Organ‐specific tumour response on first FU showed a median (IQR) shrinkage of lymph node, liver and bone metastases of 47% (30–100%), 55% (27–61%) and 39% (19–48%), respectively. Radiological response assessment according to iRECIST identified no pseudo‐progression.

**Fig. 1:**
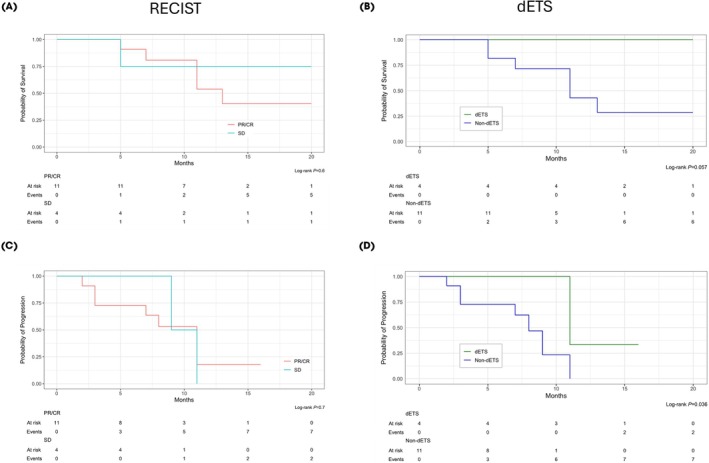
The OS and PFS stratified by response according to RECIST 1.1 (partial/complete response [PR/CR] vs stable disease [SD]) **(A, C)** and stratified by dETS **(B, D)**. While response according to RECIST 1.1 showed no prognostic value with regard to OS and PFS, dETS was associated with longer PFS.

On first imaging, dETS was observed in 25% (4/16) of patients. Time to progression was significantly longer in patients with dETS than in patients without dETS (*P* = 0.036) (Fig. 1D). Moreover, 1‐year OS was 100% among patients with dETS, compared with 43% among those who did not (*P* = 0.057; Fig. 1B). The median (IQR) FU was 14 (10–NR) months.

To the best of our knowledge, this retrospective cohort study provides the first real‐world in‐depth radiological assessment of mUTUC treated with EV + P.

Although OS and PFS intervals were shorter than in the overall EV‐302 study cohort [4], radiological response rate at the first FU imaging (69%) was comparable to the EV‐302 overall response rate (ORR) of 68%. Moreover, the ORR of our cohort exceeded both the ORR of EV‐302 (81% vs 68%) and a large meta‐analysis [4, 6]. These findings complement other real‐world mUTUC data showing high ORR in patients with mUTUC treated with EV after pembrolizumab in further line but no benefit in OS [7].

Although nearly all patients in our cohort and over 86% in the EV‐302 demonstrated disease control (complete response, partial response, stable disease) on imaging, not all patients ultimately benefited from therapy. While RECIST‐defined categories remain the standard for guiding treatment decisions in clinical practice, their prognostic value in mUTUC appears limited. Our findings suggest that static morphological evaluation may not reliably distinguish transient from durable benefit. Integrating dynamic imaging metrics such as dETS could therefore improve treatment guidance.

Across solid tumour types, image‐based markers including TB, depth of response, early tumour shrinkage, and organ‐specific response have been evaluated as prognostic indicators, but have not yet been systematically studied in mUTUC. In our study, dETS capturing both timing and magnitude of tumour reduction was prognostic for PFS, suggesting that early and substantial tumour regression is crucial for durable benefit from EV + P.

While novel biomarkers such as circulating tumour DNA or tumour cells, serum lactate dehydrogenase, or positron emission tomography/CT‐based imaging are under investigation [8], these remain limited by cost, accessibility, and standardisation. In contrast, imaging‐derived parameters such as dETS represent a readily available, cost‐effective, and reproducible tool that can be readily implemented in clinical practice to refine early treatment evaluation.

In summary, while RECIST 1.1‐based response was not prognostic, dETS emerged as a practical early‐on predictor of efficacy in mUTUC under EV + P. Validation in larger cohorts and integration with molecular markers may further enhance prognostic accuracy and support individualised treatment strategies.

## Author Contributions

Marie Semmler and Maurice Heimer: conceptualisation, data curation, formal analysis, methodology, writing – original draft preparation. Lennert Eismann: conceptualisation, formal analysis, project administration, supervision, visualisation, writing – review and editing. Philipp M. Kazmierczak: conceptualisation, writing – review and editing. Can Aydogdu, Benazir Enzinger, Isabel Brinkmann, Frederik Kolligs, Gerald B. Schulz, Christian G. Stief, Jozefina Casuscelli: data curation, writing – review and editing. All authors revised the paper and approved the final version.

## Disclosure of Interests

Marie Semmler reports meeting attendance and travel support from BMS, Janssen, MSD and Merck, and honoraria for lectures from Astellas. All other authors declare no conflict of interest.

## Funding

None.
